# Antiepileptic Drug Withdrawal in Dogs with Epilepsy

**DOI:** 10.3389/fvets.2015.00023

**Published:** 2015-08-10

**Authors:** Felix Kaspar Gesell, Sonja Hoppe, Wolfgang Löscher, Andrea Tipold

**Affiliations:** ^1^Department of Small Animal Medicine and Surgery, University of Veterinary Medicine Hannover, Hannover, Germany; ^2^Department of Pharmacology, Toxicology, and Pharmacy, University of Veterinary Medicine Hannover, Hannover, Germany

**Keywords:** seizures, discontinuation of antiepileptic treatment, risks

## Abstract

Epilepsy is one of the most common neurological disorders in dogs and is treated by chronic administration of antiepileptic drugs (AEDs). In human beings with epilepsy, it is common clinical practice to consider drug withdrawal after a patient has been in remission (seizure free) for three or more years, but withdrawal is associated with the risk of relapse. In the present study, the consequences of AED withdrawal were studied in dogs with epilepsy. Therefore, 200 owners of dogs with idiopathic or presumed idiopathic epilepsy were contacted by telephone interview, 138 cases could be enrolled. In 11 cases, the therapy had been stopped after the dogs had become seizure free for a median time of 1 year. Reasons for AED withdrawal were appearance or fear of adverse side effects, financial aspects, and the idea that the medication could be unnecessary. Following AED withdrawal, four of these dogs remained seizure free, seven dogs suffered from seizure recurrence, of which only three dogs could regain seizure freedom after resuming AED therapy. Due to the restricted case number, an exact percentage of dogs with seizure recurrence after AED withdrawal cannot be given. However, the present study gives a hint that similar numbers as in human patients are found, and the data can help owners of epileptic dogs and the responsible clinician to decide when and why to stop antiepileptic medication.

## Introduction

Epilepsy is one of the most common neurological disorders in dogs (1), characterized by spontaneous recurrent seizures originating in the central nervous system (2). The prevalence of canine epilepsy varies in different studies between 0.5% and 5% (1). If no underlying cause except of hereditary factors are found for the recurrent seizures in the diagnostic work-up, epilepsy is classified as idiopathic (3). Most canine patients that suffer from idiopathic epilepsy experience their first seizure event at an age of 1–5 years (4).

As in human beings, dogs with epilepsy are typically treated with antiepileptic drugs (AEDs) and about 40% become seizure free (1). Epilepsy can also remit spontaneously (5). The risk of recurrence of seizures after AED withdrawal in dogs that became seizure free during treatment is not known. There are different reasons to discontinue treatment with AEDs in dogs, including medical causes or owners’ perception. Some owners may not accept long-term or lifelong epilepsy treatment and as a consequence may even consider euthanasia as an alternative to treatment (1). Financial aspects could be another reason to withdraw AEDs after a seizure-free period. A better knowledge of the possible outcome in such cases could help owners and responsible clinicians to calculate the potential risk of recurring seizures and to find the right decision concerning treatment discontinuation. In human patients, behavioral and cognitive side effects are well-known adverse events in patients treated with AEDs and are shown to improve after drug tapering (6, 7). Other aspects for the decision of treatment discontinuation in human beings include the risk of teratogenicity, drug interactions with concurrent medications, and the concern that treatment might be unnecessary (8). The last reason might reflect owners perception in veterinary medicine. Withdrawal of AEDs in dogs may be attempted after a seizure-free period for >1 year but the risk of seizure recurrence has to be considered (1).

In human studies, the probability of relapse after planned discontinuation of treatment has varied between 12% and 66% in different studies (9). The chance of AED withdrawal without relapse was greater in patients with a seizure-free period on therapy of 2–5 years, a single reported seizure type, a normal neurological exam, and a normalized EEG under treatment (10). But apart from clinical experience, no clear guidelines are given in order to answer the question of the right timing and safety of AED withdrawal (11) and insufficient evidence exists to guide the timing of withdrawal of AEDs in seizure-free adults. In children, there is evidence to support waiting for at least two seizure-free years before discontinuing AEDs (12).

The hypothesis of this study was that only a part of canine patients will remain seizure free after AED withdrawal. Therefore, the outcome of a group of dogs with idiopathic or presumed idiopathic epilepsy that had discontinued antiepileptic treatment was investigated.

## Materials and Methods

The study was designed as a retrospective study using data of patients with epilepsy examined at the Department of Small Animal Medicine and Surgery of the University of Veterinary Medicine Hannover. The study was performed according to the ethical rules of the University. A thesis committee approved the study; the owners gave their written consent to use the data for a scientific study. Before the telephone interview, the owners were informed about the purpose of the study. The diagnosis or presumed diagnosis of idiopathic epilepsy was made based on typical history (4) and normal routine clinical and neurological examination. No abnormalities on hemogram or blood chemistry profile were detected. If magnetic resonance imaging (MRI) and cerebrospinal fluid (CSF) evaluation were performed, no abnormalities were detected (Table 1).

**Table 1:** **Overview of the patients included into the study; n.d., not done; seizure type, loss of consciousness during the seizure, occurrence of cluster seizures prior to antiepileptic drug withdrawal; m, male, f, female, c, male castrated**.

Patient	Age at seizure onset, sex	MRI/cerebrospinal fluid	Primary seizure type	Loss of consciousness during the seizure	Seizure cluster	Therapy	Seizure-free period before withdrawal	Recurrence of seizures after withdrawal	Follow-up period after withdrawal	Seizure freedom after new therapy attempt
1	9 years, m	Normal findings	Generalized convulsive	Yes	No	Phenobarbital	3 months	No	6.5 years	No treatment necessary
2	3 months, m	Normal findings	Generalized convulsive	No	Yes	Potassium bromide	9 months	No	7 years	No treatment necessary
3	6 months, m	n.d.	Generalized convulsive	No	Yes	Phenobarbital	5 years	No	2 years	No treatment necessary
4	2 years, m	n.d.	Generalized convulsive	Yes	No	Phenobarbital	1 year	No	6 years	No treatment necessary
5	4 years, m	n.d.	Generalized convulsive	No	Yes	Phenobarbital	Several months	Yes	7 years	No restart of therapy
6	2 years, c	Normal findings	Generalized convulsive	Yes	No	Phenobarbital, zonisamide	4 years	Yes	4 years	No restart of therapy
7	1.5 years, m	n.d.	Generalized convulsive	No	Yes	Phenobarbital	1 year	Yes	5 years	No
8	2 years, m	Normal findings	Generalized convulsive	Yes	Yes	Phenobarbital	4 years	Yes	5.5 years	No
9	3 years, f	Normal findings	Focal	No	Yes	Imepitoin	1 year	Yes	2 years	Yes
10	2.5 years, m	n.d.	Generalized convulsive	Yes	Yes	Phenobarbital	1.75 years	Yes	0.5 year	Yes
11	7 years, m	n.d.	Focal	No	No	Phenobarbital	2 years	Yes	5 years	Yes

The owners of dogs with idiopathic or presumed idiopathic epilepsy were contacted by telephone calls. Follow-up information was gathered by the use of a questionnaire, including age at first seizure event, the predominant seizure type, occurrence of cluster seizures, treatment, and eventual AED withdrawal. In cases with discontinuation of treatment with AEDs, it was noted whether seizures recurred and differed from the previous seizure types. Furthermore, questions were included to note, if these recurrent seizures could be controlled again with a new treatment attempt (see telephone questionnaire in Supplementary Material).

A total of 200 cases with idiopathic or presumed idiopathic epilepsy were collected for the telephone interview. In 62 of these 200 cases, either the owners could not be reached because of missing contact details or no information about the dog was available. In 11 of the 138 evaluated patients, the AEDs were withdrawn after the dogs became seizure free for a certain time period (Table 1).

## Results

In 11 (8%) of the 138 cases with successfully completed telephone interviews, the AEDs, in most cases phenobarbital, had been withdrawn (Table 1). The withdrawal was conducted in a controlled way after step by step reduction of the AED dosage over a required time period, but varied widely between about 1 and 3 months according to the recommendation of the private practitioner. Of these patients, 10 dogs were male (nine intact, one neutered) and one dog was female.

Except one dog that received phenobarbital and zonisamide, all patients with AED withdrawal had received only one AED. Eight of these patients were treated with phenobarbital prior to withdrawal, one dog with potassium bromide, and one dog with imepitoin (13). Serum drug concentrations were not available in all dogs at the timepoint of drug withdrawal.

Eight dogs (72.7%) of these patients had received the drugs for at least a minimum of 1 year prior to drug withdrawal. One dog had received treatment for only 3 months and another for 9 months. In one case, the owner only estimated the time period under treatment with several months. The maximum of the time period under treatment had been 5 years prior to drug withdrawal. Median time of treatment prior to withdrawal was 1 year. The medication of each dog is shown in Table 1.

The reasons for the owners to withdraw the AEDs had been appearance or fear of adverse side effects (two dogs), financial burden (one dog), and/or the feeling or the desire that AEDs could be unnecessary (eight dogs).

Four (36.4%) of the 11 dogs with withdrawal of antiepileptic treatment after a seizure-free period remained seizure free. The follow-up period after the withdrawal of the AED for these dogs was between 2 and 7 years (2, 6, 6.5, and 7 years). Seven dogs (63.6%) showed recurrence of seizures after withdrawal. Their primary seizure type before AED withdrawal, loss of consciousness during the seizure, or appearance of cluster seizures is shown in Table 1. Of these patients, three dogs (42.8%) became seizure free again after restarting antiepileptic treatment. The follow-up period after the new therapy attempt was between 0.5 and 5 years. In two dogs (25.6%), seizures could not be controlled again. One of these patients had to be euthanized because of recurrent seizures resistant to a new therapy attempt on the request of the owner. The other one was treated with the same medication (phenobarbital) as given before AED withdrawal and with potassium bromide as an add-on treatment. The follow-up period for this dog after recurrence of seizures was 5 years. In this patient, the seizure type was equal to the seizures before withdrawal and seizure frequency was low (one seizure/3 months). In two cases (25.6%), the owners did not try a new antiepileptic treatment because the frequency of the recurrent seizures was much lower and the owners were satisfied with this outcome.

## Discussion

In most cases, the therapy of an epileptic dog involves a long-term or even lifelong treatment with AEDs (2, 3). However, the duration of antiepileptic treatment is sometimes decided in an individual way for each patient. The decision of discontinuing AEDs is a question facing both the owner of an epileptic dog and the responsible clinician. In human medicine, there are some known factors that increase the risk of seizure recurrence after AED withdrawal. Risk factors for seizure recurrence are an adolescent-onset epilepsy, a longer duration of epilepsy, occurrence of partial seizures, an abnormal neurological examination, and an abnormal EEG (8–10). In veterinary medicine, the owners may ask for withdrawal of AEDs for different reasons. In our study, these reasons were the appearance or fear of adverse side effects, the financial burden, and/or the feeling that AEDs could be unnecessary. But as in human beings, the withdrawal of AEDs in the canine patient is a well-known risk and even after a seizure-free period the patient might develop recurrent seizures after drug withdrawal as shown in the current study. Furthermore, the seizures may appear more frequently and less controllable after tapering AEDs. In a review of the clinical literature, Schmidt and Löscher (9) found that between 12% and 66% of seizure-free human patients relapsed after planned discontinuation of AEDs. Reinstitution of AEDs after recurrence was efficacious in only 80% (range 64%–91%) of the patients (9). Factors associated with poor treatment outcome of treating recurrences were symptomatic etiology, partial epilepsy, and cognitive deficits. In the canine patients, similar studies are not available to the best of the author’s knowledge, and no other guidelines than personal clinical experience of the veterinarian are considered for prognosis and outcome estimation.

In the current study, in 11 of 138 dogs with idiopathic or presumed idiopathic epilepsy AEDs had been withdrawn. Albeit a large number of dogs with long-term antiepileptic treatment were evaluated, medication was stopped only in a small part of these dogs. As already reported in the literature for human patients (9), some of these dogs suffered from seizure recurrences, others remained seizure free. The distribution was 36.4% dogs remaining seizure free and 63.6% with recurrence of seizures reflecting a high rate of seizure recurrence. On the other hand, this result supports the common opinion that withdrawal of antiepileptic treatment after a seizure-free period might be successful in at least a part of the patients. But as shown by our study, it is very important to control the patients in the time of withdrawal because seizure recurrence is a risk that should not be underestimated.

Recurring seizures after AED withdrawal can be divided into recurrence of the original epilepsy, a drug withdrawal (“rebound”) phenomenon or drug resistance that evolved during the drug discontinuation (9). In our cases, it is not possible to determine exactly the underlying type because in the most cases the new therapy attempt started immediately after the recurrence of new seizure events. In those cases where the owners did not want to try a new therapy attempt, it was probably not just a drug withdrawal phenomenon associated with physical dependence during treatment (see below) because the seizures remained present in these dogs for the rest of their lives.

In three of the seven patients with seizure recurrence, seizures were already observed during the time of withdrawal. In another patient, the seizures occurred 1 month after withdrawal. Therefore, the time interval for a higher risk of seizure recurrence seems to be during the withdrawal time itself and in the immediate time afterward. Most epileptic dogs are treated with phenobarbital, which is one of the few AEDs approved for treatment of epilepsy in dogs, but leads to development of physical dependence and the risk of withdrawal seizures upon termination of treatment (14). Dogs should be carefully controlled and observed when the owner decides to withdraw the AEDs in order to recognize and treat potential seizures.

The decision to withdraw AEDs might also be affected by the risk of occurrence of refractory seizures. In human being, the reinstitution of AEDs after recurrent seizures after drug withdrawal resulted in seizure remission in 64%–91% (8). In our study, the owners of two patients did not want to try a new treatment attempt. Three of the other five patients were seizure free again after restarting AEDs. Of the remaining two cases, one dog was euthanized due to uncontrolled recurrent seizures. The other one responded to the treatment, but was not seizure free anymore. Therefore, the owners have to be aware that eventually recurrent seizures following AED withdrawal may be less controllable to AEDs.

In the current study, 11/138 cases with AED withdrawal were investigated. Most of the contacted owners did not discontinue the medication and seemed to be satisfied with the result of achieving seizure freedom or reduced seizure frequency with a lifelong therapy. Despite the limited number of patients, our study shows that different outcomes after drug withdrawal are to be expected. Besides a good prognosis or outcome, seizure recurrence was observed in the withdrawal period itself and the immediate months afterward. A good compliance is absolutely necessary when trying to discontinue AEDs in dogs with epilepsy. Owners should control their dogs very carefully in this time period in order to recognize signs of seizure recurrence as fast as possible.

## Conflict of Interest Statement

None of the authors have a financial or personal relationship with other persons or organizations that could inappropriately influence or bias the content of the paper.

## Supplementary Material

The Supplementary Material for this article can be found online at http://journal.frontiersin.org/article/10.3389/fvets.2015.00023
