# Supplementary material for: Antiepileptic Drug Withdrawal in Dogs with Epilepsy
Source: Front Vet Sci. 2015 Aug 10;2:23. doi: 10.3389/fvets.2015.00023 (PMC4672179; doi:10.3389/fvets.2015.00023)
Supplement: Supplementary file 1 [file Data_Sheet_1.DOCX]

Telephone Questionnaire:

Age of first seizure event

Seizure frequency

Characterisation of seizures

Duration of seizures

Cluster seizures

Special events preceding seizures (reactive seizures, trauma)

Diagnostic workup

Antiepileptic treatment

Seizures during antiepileptic treatment

When dog became seizure free – timepoint since last seizure event

Antiepileptic treatment without or with discontinuation

When discontinuation – why was the treatment terminated

How was the withdrawal performed – with or without tapering the dosage

Seizure events after antiepileptic drug withdrawal

Characterisation of seizure events after antiepileptic drug withdrawal

Did seizure events change in appearance after withdrawal (intensity, frequency, duration)

New antiepileptic treatment after withdrawal

Others (time for owners at the end of the telephone interview to talk about their dog)
